# Analysis of the *WUSCHEL*-*RELATED HOMEOBOX* gene family in the conifer *picea abies* reveals extensive conservation as well as dynamic patterns

**DOI:** 10.1186/1471-2229-13-89

**Published:** 2013-06-12

**Authors:** Harald Hedman, Tianqing Zhu, Sara von Arnold, Joel J Sohlberg

**Affiliations:** 1 Department of Plant Biology and Forest Genetics, Uppsala BioCenter, Swedish University of Agricultural Sciences and Linnean Center for Plant Biology, PO-Box 7080, Uppsala, SE, 75007, Sweden

**Keywords:** *Picea abies*, Somatic embryogenesis, *WOX* genes

## Abstract

**Background:**

Members of the *WUSCHEL*-*RELATED HOMEOBOX* (*WOX*) gene family have important functions during all stages of plant development and have been implicated in the development of morphological novelties during evolution. Most studies have examined the function of these genes in angiosperms and very little is known from other plant species.

**Results:**

In this study we examine the presence and expression of *WOX* genes in the conifer *Picea abies*. We have cloned 11 *WOX* genes from both mRNA and genomic DNA and examined their phylogenetic relationship to *WOX* genes from other species as well as their expression during somatic embryogenesis and in adult tissues.

**Conclusions:**

Our study shows that all major radiations within the *WOX* gene family took place before the angiosperm-gymnosperm split and that there has been a recent expansion within the intermediate clade in the Pinaceae family. Furthermore, we show that the genes from the intermediate clade are preferentially expressed during embryo development in *Picea abies*. Our data also indicates that there are clear orthologs of both *WUS* and *WOX5* present in the *P*. *abies* genome.

## Background

The morphogenesis of the plant body is a developmental process that continues throughout the entire lifespan of the plant. Different morphologies in different plant lineages are dependent on developmental differences that have a molecular basis in the function and/or expression of regulatory genes. In seed plants major patterning events take place during embryo development, the embryo is polarised with an apical shoot meristem, giving rise to all above ground parts, and a basal root meristem, giving rise to the root system. The specification of the body plan during embryogenesis requires the coordination of cell fates according to their position along the embryo axis, from the apical to the basal part. During post-embryonic development new organs are formed successively from the shoot- and root meristems at the same time as the meristems are maintained. The genetic control of patterning and morphogenesis during plant development is dependent on a large number of genes. Many aspects of the molecular regulation of plant development have been analysed, primarily in the model angiosperm species *Arabidopsis thaliana*, whereas very little is known in plant species outside the angiosperms.

In angiosperms members of the *WUSCHEL*-*RELATED HOMEOBOX* (*WOX*) gene family play important roles determining cell fates during plant development. The *WOX* gene family is characterised by the phylogenetic relationship of the homeodomain of these genes. It is present only in the “green” lineage comprising land plants and green algae. The number of *WOX* genes present in the genomes of different species correlates to some degree with the complexity of the species body pattern. Only one *WOX* gene is found in the genomes of the green micro algae *Ostreococcus tauri* and *O*. *lucimarinus*, three *WOX* genes are present in the genome of the moss *Physcomitrella patens*, nine in the lycophyte *Selaginella moellendorffii*, whereas the genome of *A*. *thaliana* contains 15 *WOX* genes. Phylogenetic analyses have divided the *WOX* gene family into three major clades [1-3]. Only one clade contains genes from early diverging plants, e.g. the moss *P*. *patens*, as well as the green algae *O*. *tauri* and *O*. *lucimarinus*, and this clade is therefore referred to as the ancient clade [1]. The ancient clade also contains representatives from *A*. *thaliana*, namely *AtWOX10*, *13*, and *14*. The other two clades are termed the intermediate clade, containing *AtWOX8*, *9*, *11* and *12*, and the modern clade, containing *AtWUS* and *AtWOX1*-*7*[1].

The role of the *WOX* genes during plant development has been studied to some detail in *A*. *thaliana* as well as in *Petunia hybrida*, *Zea mays* and *Oryza sativa* (e.g. [4-7]). The founding member of the gene family, *A*. *thaliana WUSCHEL* (*AtWUS*), has been shown to act in the organising centre of the shoot apical meristem (SAM) to maintain the stem cell population [4,8]. A similar role has been proposed for *AtWOX5* in the root apical meristem [9] and for *AtWOX4* in the cambial meristem [10,11]. Other *WOX* genes have been implicated in the patterning and morphogenesis of the early embryo, e.g. *AtWOX2*, *AtWOX8* and *AtWOX9*[12-14] and in regulating flower development and/or inflorescence architecture, e.g. *AtWOX1*, *AtWOX3*, *AtWOX6* and *P*. *hybrida EVERGREEN* (*PhEVG*) and *SISTER OF EVERGREEN* (*PhSOE*) [5]. The *WOX* genes function as transcriptional regulators and at least some can act as both activators and repressors depending on tissue type or developmental stage [15]. Furthermore, all *WOX* genes examined show very specific expression patterns, both spatially and temporally, which are important for their functions (e.g. [16]). Several studies suggest that the *WOX* gene family may be involved in the evolution of developmental processes [3,5,17,18]. Thus, analysis of the tissue specific expression of *WOX* genes is of interest to elucidate similarities and differences in the regulatory mechanisms of plant development also in species outside the angiosperms.

The *WOX* genes in *O*. *tauri* (*OtWOX*) and *P*. *patens* (*PpWOX01*-*03*), all belonging to the ancient clade, have been shown to be constitutively expressed in all tissues and at all developmental stages analysed [3]. The *A*. *thaliana* ancient clade genes *AtWOX13* and *AtWOX14* are also expressed in most tissues and developmental stages (roots, shoots and reproductive organs), although the expression pattern is limited to certain cells within an organ [3]. In the conifer *Picea abies* the intermediate clade gene *PaWOX8*/9 has been shown to be preferentially expressed during embryo development [19]. Furthermore, the *P*. *abies* modern clade gene *PaWOX2* was shown to have a similar expression pattern as *PaWOX8*/*9*[19,20]. The *A*. *thaliana* genes *AtWOX8* and *AtWOX9*, which belong to the intermediate clade are expressed in specific domains from the first cell division of the proembryo until the end of embryogenesis and the same is true for the *A*. *thaliana* modern clade gene *AtWOX2*[13]. In the fern *Ceratopteris richardii* it has been shown that *CrWUL*, a gene with strong similarity to *AtWUS*, is expressed in the shoot meristem [21]. Another *AtWUS* homolog, *GgWUS* from the gymnosperm *Gnetum gnemon*, is expressed in both the root and shoot meristems. Furthermore, heterologous expression of *GgWUS* in *A*. *thaliana* confers the same phenotype as the overexpression of *AtWUS*[2]. Thus, although little is known about the *WOX* genes outside the angiosperms most studies have revealed extensive similarities, at least with regard to gene expression, suggesting a conserved function for these genes. However, the shoot specific expression of *WUS* and root specific expression of *WOX5* seem to be specific to angiosperms as the gymnosperm *WUS* homologs *GgWUS*, *Ginkgo biloba WUS* (*GbWUS*), and *Pinus sylvestris WUS* (*PsWUS*) were shown to be expressed in both the shoot and the root [2]. Furthermore, Nardmann et al. (2009) could only detect one homolog of *WUS*/*WOX5* in these gymnosperms suggesting that the *WUS* and *WOX5* genes are the result of an angiosperm specific gene duplication [2].

In this paper we present an analysis of the *WOX* gene family in the conifer *P*. *abies*. We have identified 11 *WOX* homologs and analysed the phylogenetic relationship of these genes to other known *WOX* genes. Our phylogenetic analyses have identified one member of the ancient clade and several members in the intermediate and modern clades. The *P*. *abies WOX* genes of the intermediate clade group together apart from the angiosperm genes, whereas there are clear *P*. *abies* orthologs of most angiosperm modern clade genes. Furthermore, we have analysed the expression of the different *WOX* genes in different tissues and developmental stages with a special focus on somatic embryo development.

## Results

### Cloning and phylogeny of *P. abies WOX* genes

**Figure 1:**
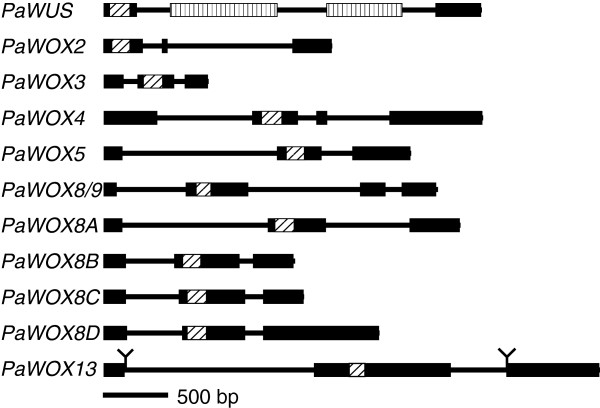
**Gene structure of *P. abies WOX *genes.** Exons are thick and black, introns are thin lines. The conserved homeobox region is striped diagonally. The repeat regions in *PaWUS* are striped vertically. The gene structure of *PaWOX13* is only putative. Due to long introns exon 1 and 3 are derived from cDNA sequence data only. End of known intron sequences are marked with lines.

We have successfully isolated 11 *P*. *abies WOX* genes using degenerate primers targeting the homeodomain. The isolated homeodomain sequences were extended by genome walking to acquire the full genomic sequences (exons and introns). Each genomic locus was then cloned using gene specific primers to confirm the genome walking and the exon-intron pattern was predicted. The gene structure of the isolated genes is presented in Figure 1. Two of these genes have been described as cDNAs earlier, namely *PaWOX2* and *PaWOX8*/*9*[19,20]. The *P*. *abies WOX* genes were named according to their closest homolog in *A*. *thaliana* as indicated by phylogenetic analyses (see below) except for *PaWOX8A*, *PaWOX8B*, *PaWOX8C*, and *PaWOX8D*, which were named after *AtWOX8*, which is the *A*. *thaliana* gene in the intermediate clade with the lowest number, and *A*-*D* because the *P*. *abies* genes are more similar to each other (65% average pairwise identity, also see Additional file 1) than they are to any *A*. *thaliana* genes. Due to long introns we were not able to amplify the full-length genomic sequence of *PaWOX13* by either genome walking or with gene specific primers, thus the gene model is made from a combination of genomic and transcript data and lacks sequence in both introns (Figure 1).

We have cloned transcripts containing a full coding sequence for all genes except for *PaWUS*, *PaWOX8C* and *PaWOX8D* for which we could not detect any transcripts in the tissues analysed. According to our predictions these latter three genes have intact open reading frames (ORFs), although the predicted *PaWUS* intron is more than 2 kb and contains repetitive sequences (Figure 1). We were not able to detect any transcript of *PaWUS* using primers targeting either the homeobox alone or targeting the entire ORF.

**Figure 2:**
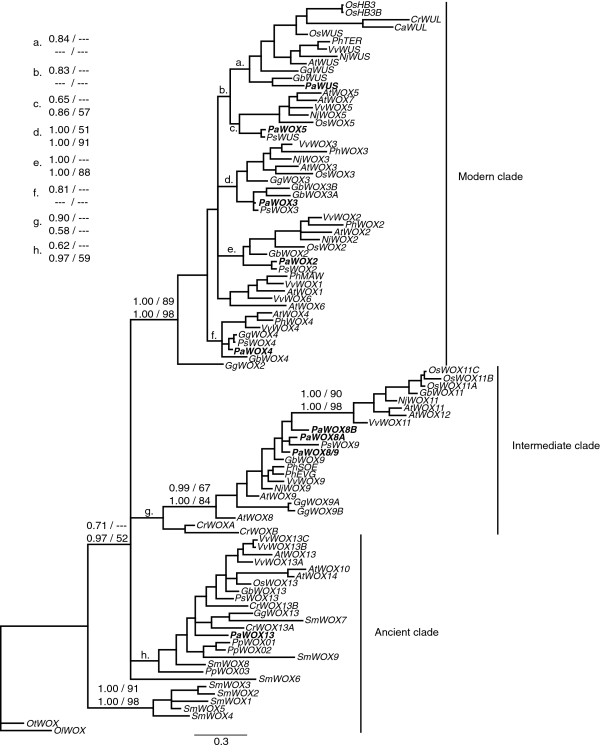
**Phylogenetic tree of the *WOX *genes.** Support values indicate: posterior probability using DNA data/bootstrap support using DNA data, on the top row and: posterior probability using protein data/bootstrap support using protein data, on the lower row. Support values are indicated on the respective branch except when the space is too limited in which case a letter indicates the branch. The tree shown was made using Bayesian inference and DNA data. The tree contains sequences from green algae (*Ostreococcus tauri*, *Ot* and *O*. *lucimarinus*, *Ol*), bryophytes (*Physcomitrella patens*, *Pp*), lycophytes (*Selaginella moellendorffii*, *Sm*), ferns (*Ceratopteris richardii*, *Cr* and *Cyathea australis*, *Ca*), gymnosperms (*Ginkgo biloba*, *Gb*, *Gnetum gnemon*, *Gg*, *P*. *abies*, *Pa*, and *Pinus sylvestris*, *Ps*), and angiosperms (*A*. *thaliana*, *At*, *Nymphaea jamesoniana*, *Nj*, *Oryza sativa*, *Os*, *Petunia hybrida*, *Ph*, and *Vitis vinifera*, *Vv*). The *P*. *abies* sequences are in bold.

**Figure 3:**
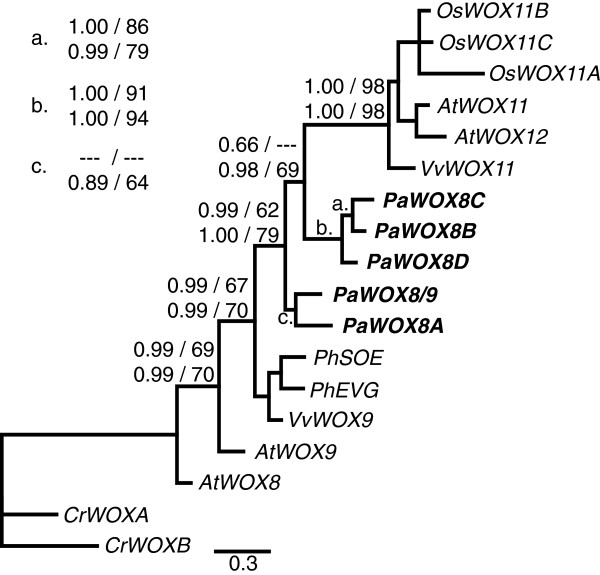
**Phylogenetic tree of the intermediate clade.** Support values and species abbreviations are indicated as in Figure 2. The tree shown was made using Bayesian inference and protein data. *P*. *abies* sequences are in bold.

**Figure 4:**
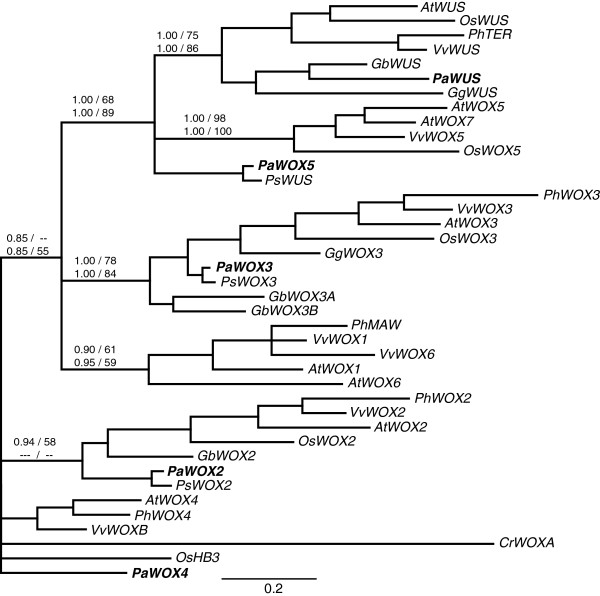
**Phylogenetic tree of the modern clade.** Support values and species abbreviations are indicated as in Figure 2. The tree shown was made using Bayesian inference and protein data. *P*. *abies* sequences are in bold.

To put the *P*. *abies WOX* genes in a phylogenetic context the homeodomain sequences from the *P*. *abies WOX* genes were aligned to a subset of homeodomain sequences from other *WOX* genes. We included sequences from green algae, bryophytes, lycophytes, ferns, gymnosperms, and angiosperms and used two different phylogenetic methods and both nucleotide and protein alignments to infer the phylogeny. The analyses showed good support for the three major clades of *WOX* genes, the ancient clade, intermediate clade, and modern clade (Figure 2). However, there was some uncertainty as to the placement of the fern sequences *CrWOXA* and *CrWOXB* in the intermediate clade since the support is quite low, especially in the Maximum Likelihood (ML) analyses. According to the phylogenetic analysis there was one *P*. *abies* representative in the ancient clade, *PaWOX13*, and several representatives of the intermediate clade (*PaWOX8*/*9* and *PaWOX8A*-*D*) and the modern clade (*PaWOX2*-*5*, *PaWUS*) (Figure 2). In order to be able to elucidate the relationships in the intermediate and modern clades in more detail new sub-trees including only members from the respective clades were generated using additional sequence outside the homeodomain (Figures 3 and 4).

In the intermediate clade there were no clear *P*. *abies* orthologs to the angiosperm sequences (Figure 3). The ML analysis using nucleotide sequences supported a monophyletic group for the *P*. *abies* genes (data not shown) whereas the other methods suggested a division into two sub-clades. There was good support for the monophyly of the genes in the clade containing *AtWOX11* and *12* (Figure 3). However, the *AtWOX8* and *9* genes, as well as the *PhEVG*, *PhSOE* and *VvWOX9* genes, did not show a monophyletic relationship. It is therefore difficult, with this limited data set, to conclude if the *P*. *abies* genes are more closely related to *AtWOX11* and *12*, closer to *AtWOX8* and *9*, or if they should group as sister to the angiosperm sequences.

In the modern clade there was almost a complete set of orthologous sequences to the *A*. *thaliana* sequences (Figure 4). The support was good for the orthology of *PaWUS* and *AtWUS*, as well as for *PaWOX3* and *AtWOX3*. The support was not very strong, but the topology and number of genes still support the orthology of *PaWOX2* and *AtWOX2*, *PaWOX4* and *AtWOX4*, and *PaWOX5* and *AtWOX5*. The only modern clade *A*. *thaliana* sequences we did not find orthologs for in *P*. *abies* were *AtWOX1*, *AtWOX6* and *AtWOX7*. The support for the orthology of *PaWOX2* and *AtWOX2* was rather strong in the full *WOX* gene tree (Figure 2) but not as strong in the modern clade sub-tree (Figure 4). The monophyly of the *WOX4* clade had a low support in the full *WOX* gene tree (Figure 2). In the modern clade sub-tree there was no support for the monophyly of the *WOX4* genes. Although the monophyly is in question *PaWOX4* still groups closer to *AtWOX4* than to any other *A*. *thaliana WOX* gene. The support for *PaWOX5* to group with *AtWOX5* was not very strong (Figures 2 and 4). However, there was good support for the *WUS*/*WOX5* sub-clade (Figure 4) including all *WUS* and *WOX5* orthologs from the different species.

### Expression of *WOX* genes in *P. abies*

**Figure 5:**
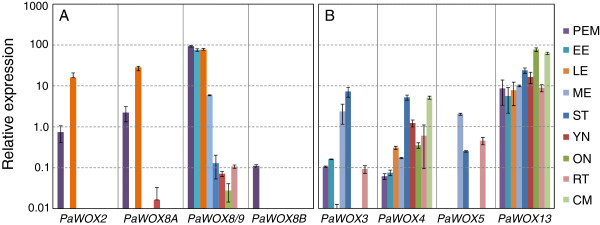
**Quantitative real-time PCR of *P*. *abies WOX *gene expression in different tissues (±SEM). A**. Relative expression of *PaWOX2*, *PaWOX8*/*9*, *PaWOX8A* and *PaWOX8B*. **B**. Relative expression of *PaWOX3*, *PaWOX4*, *PaWOX5* and *PaWOX13*. The Y-axis is presented as natural logarithm. Absence of staples indicates no expression detected. PEM, proembryogenic masses; EE, early embryos; LE, late embryos; ME, mature embryos; ST, shoot tips; YN, young needles; ON, old needles; RT, root tips; CM, cambium.

In order to get a better understanding of where the *P*. *abies WOX* genes are active we analysed their expression in different tissues and at various developmental stages using quantitative real-time PCR (qPCR) (Figure 5). *PaWOX8*/*9* was mainly expressed during embryo development although some expression could be detected outside the embryo at very low levels (Figure 5A). *PaWOX2* and *PaWOX8A* showed a very specific expression pattern with expression only detectable in proembryogenic masses (PEMs) and late embryos (Figure 5A). No expression was detected in early or mature embryos. *PaWOX8A* expression could also be detected in young needles although this expression was extremely low. Thus, *PaWOX2*, *PaWOX8A* and *PaWOX8*/*9* are all more or less embryo specific. PEMs were the only tissue where we could detect expression of *PaWOX8B* (Figure 5A). The high sequence similarity between *PaWOX8B*, *C*, and *D* made it very difficult to design gene specific primers, therefor the expression we detected could potentially also include *PaWOX8C* and *D* and not only *B*. However, we cloned and sequenced several qPCR products (16) and they all corresponded to *PaWOX8B*.

The highest expression of *PaWOX3* was detected in mature embryos and shoot tips whereas a very low expression was detected in PEMs, early embryos and root tips (Figure 5B). *PaWOX4* showed expression in all tissues analysed with a maximum in shoot tips and cambium (Figure 5B). *PaWOX5* expression was only detected in mature embryos, shoot tips and root tips (Figure 5B). Interestingly, *PaWOX5* was the only gene not expressed in PEMs. *PaWOX13* expression was detected in all tissues analysed (Figure 5B). The expression was somewhat higher in old needles (last years shoot) and in cambial tissue whereas the expression levels in other tissues seemed to be very similar.

## Discussion

Previous studies of the *WOX* gene family in plants have identified three major clades; the ancient clade with representatives from all lineages of land plants as well as green algae, and the intermediate and modern clades with representatives found only in ferns and seed plants [1-3,21]. In our study we have identified 11 *P*. *abies WOX* genes representing all major clades of *WOX* genes. Only one *P*. *abies* gene groups into the ancient clade (Figure 2), *PaWOX13*, which is in accordance to what has been reported for other gymnosperms (*G*. *gnemon*, *G*. *biloba*, and *P*. *sylvestris*) [2]. At this point we cannot exclude the existence of additional genes of the ancient clade in gymnosperms. We could detect expression of *PaWOX13* in all tissues tested, both during embryogenesis and in more adult tissues. The ancient clade genes *AtWOX13* and *AtWOX14* from *A*. *thaliana* are expressed in most tissues, although there is no clear expression data from the embryo stage [3]. Thus at this scale of resolution the expression patterns of the ancient clade genes are similar in *P*. *abies* compared to *A*. *thaliana*, i.e. a general expression in most tissues.

We could identify five different genes belonging to the intermediate clade (*PaWOX8*/*9* and *PaWOX8A*, *B*, *C*, and *D*). The phylogenetic analyses show that these genes group very closely even though there is not enough support to confirm if they are monophyletic or not (Figure 3). Several of the *P*. *abies* intermediate clade genes are very similar to each other, i.e. *PaWOX8B*-*D*, especially in the coding regions (Additional file 1), suggesting that they are the result of very recent gene duplications. We could identify at least two different sequence variants of each gene, which we believe are allelic (data not shown). Therefore we cannot exclude the existence of more intermediate clade genes in *P*. *abies*. Based on this we suggest that the *P*. *abies* intermediate clade genes are the result of an expansion that happened after the split between gymnosperms and angiosperms. Interestingly, we could find at least four different EST sequences from *Pinus taeda* in Genbank (accession numbers DR690333, DT636589, DR692518, DT634290) belonging to the intermediate clade. Phylogenetic analyses group these genes within the *P*. *abies* intermediate clade genes suggesting that the expansion might be common to species within the Pinaceae family (Additional file 2). Whether this is common also to other gymnosperm lineages is still an open question.

*PaWOX8A* and *PaWOX8*/*9* show expression preferentially during embryo development with only a very low expression for *PaWOX8*/*9* in non-embryo tissues. These expression patterns could be compared with those for *AtWOX8*, *9*, *11*, and *12* since the phylogenetic analyses do not conclusively show which of these *A*. *thaliana* gene(s) are the closest homologs of the *P*. *abies* genes. *AtWOX8* and *AtWOX9* are expressed during embryo development from very early stages and throughout development [13]. *AtWOX8* expression is very low outside the embryo whereas *AtWOX9* is expressed also in more adult tissues where it is involved in the maintenance of the SAM [22,23]. The expression of *AtWOX11* and *AtWOX12* has so far not been described in any detail but array data suggest that at least *AtWOX11* is expressed during embryo development (Arabidopsis eFP Browser, [24]). Thus, the expression of *PaWOX8*/9 and *PaWOX8A* seems to be comparable to the *A*. *thaliana* intermediate clade genes although their expression is more limited to embryo development. Furthermore, the before mentioned *P*. *taeda* EST sequences were all derived from embryogenic tissues, which might suggest that the embryo specific function of these genes is common within the Pinaceae family. *PaWOX8B* expression was only detected in PEMs, which is a very specific tissue type for embryogenic cell cultures and therefor we do not know if the gene has any specific function during the life cycle of the plant. We could not detect any expression of *PaWOX8C* and *D*, which might suggest that these genes are inactive pseudogenes. However, there is nothing in the genomic sequences of these loci to support this notion. Taken together, *PaWOX8B*-*D* might all be silenced, or at least partially silenced, recent copies of either *PaWOX8A* or *PaWOX8*/*9*.

Our phylogenetic analyses identified an almost complete set of orthologous sequences in the modern clade between *P*. *abies* and *A*. *thaliana*. The only *A*. *thaliana* sequences we could not find orthologs of were: *AtWOX1*, *AtWOX6* and *AtWOX7*. *AtWOX7* is most likely the result of a very recent duplication in the *A*. *thaliana* lineage since *AtWOX5* and *AtWOX7* are more closely related to each other than to *WOX5* orthologs from *O*. *sativa* or *V*. *vinifera* (Figure 4). The lack of orthologous sequences to *AtWOX1* and *6* might be because we were unable to amplify them with our primers, alternatively there has been either an angiosperm specific diversification or a gymnosperm specific gene loss.

The orthology of *PaWOX2* with *AtWOX2* is well supported in the full *WOX* tree (Figure 2) whereas the support is lower in the modern clade sub-tree (Figure 4). In *A*. *thaliana AtWOX2* is expressed during embryo development and at low levels outside the embryo [13]. This is similar to the pattern seen for *PaWOX2* (Figure 5). However, in a previous study *PaWOX2* was shown to be expressed both in embryos and seedlings [20]. We could recapitulate the results by Palovaara & Hakman [20] when using the same primers as they used whereas our primers only detected expression in PEMs and late embryos (Additional file 3). These differences might seem strange at first but the main difference between our primers and the primers of Palovaara & Hakman [20] is that one of our primers spans an exon-intron boundary and does not amplify genomic DNA or unspliced transcripts whereas the primers of Palovaara & Hakman [20] are situated within the same exon and therefore cannot discriminate between processed and unprocessed transcript or genomic DNA (Additional file 3). It will be interesting to learn if the difference between our results and those of Palovaara & Hakman (2008) is due to that *PaWOX2* is regulated post-transcriptionally.

The orthology of *PaWOX3* and *AtWOX3* is well supported (Figure 4), which is suggestive of a conserved function for these genes in the different lineages. We could detect expression of *PaWOX3* mainly in shoot tips and mature embryos, which, at this level of resolution, is similar to the expression reported for *AtWOX3* as well as for a *Z*. *mays WOX3* ortholog [25,26].

In our phylogenetic analyses the *WOX4* clade has the lowest support (Figures 2 and 4). Furthermore, we could not find a clear WUS-box in the deduced PaWOX4 protein (see Additional file 4). However, since *AtWOX4* is the closest *A*. *thaliana* homolog we named the *P*. *abies* gene in this clade *PaWOX4*. The expression of *PaWOX4* is not limited to any particular tissue types, rather we could detect expression in most tissues. Interestingly, high expression was detected in the cambium, which might suggest that the function of *WOX4* as a regulator of the cambial meristem is conserved between angiosperms and gymnosperms.

In contrast to other analysed gymnosperms (*G*. *gnemon*, *G*. *biloba* and *P*. *sylvestris*) [2] we found sequences grouping with both *WUS* and *WOX5* (Figure 4). The phylogenetic support for the *WUS* clade, including *PaWUS*, is good (Figure 4), and is further supported by the fact that the PaWUS protein sequence contains an extra amino acid residue in the homeodomain, which is specific to proteins in the *WUS* clade [2]. In angiosperm WUS proteins, as well as in PaWUS and GbWUS, this extra amino acid is a tyrosine whereas it is a histidine in GgWUS and the fern sequences CrWUL and CaWUL [21]. The PaWOX5 and PsWUS proteins on the other hand contain no extra amino acid, consistent with them not being WUS orthologs, but rather orthologous to WOX5. Thus, the results presented here together with previous analyses [2], suggest that the gene duplication leading to distinct *WUS* and *WOX5* genes predates the gymnosperm-angiosperm split. Although our data support the notion that there was both a *WUS* and *WOX5* ortholog present in the last common ancestor of angiosperms and gymnosperms it might not mean that the division of function between the apical meristems of shoots and roots was present. Expression of *NjWUS*, from the basal angiosperm *Nymphea jamesoniana*, has only been detected in the shoot [2] whereas expression of the gymnosperm genes *GgWUS* and *PaWOX5* has been detected in both shoot tips and root tips ([2] and this study). This suggests that the subfunctionalisation of *WUS* and *WOX5* took place at the base of the angiosperm lineage after the split between angiosperms and gymnosperms. It is interesting to note that in *G*. *biloba* and *G*. *gnemon* it is the *WUS* ortholog that is expressed whereas the existence of a *WOX5* ortholog is uncertain [2] and in the conifers *P*. *sylvestris*[2] and *P*. *abies* it is the *WOX5* ortholog that is transcribed and we could not find any expression of the *WUS* ortholog. Further investigations aimed at understanding the tissue specific expression and function of the gymnosperm *WUS*/*WOX5* genes might reveal specific functions for these genes in gymnosperms compared to angiosperms and if there are differences within the gymnosperms.

## Conclusions

The results presented here show that the major diversifications within the *WOX* gene family happened before the split between angiosperms and gymnosperms approximately 300 million years ago. Together with previous studies it confirms the dynamic patterns and lineage specific evolutionary trajectories within certain branches of the *WOX* gene tree. First, it shows that there has been an independent expansion of the intermediate clade in the Pinaceae family. Second, it shows that there are clear orthologs of both *WUS* and *WOX5* present in the gymnosperm *P*. *abies*. Thus, further investigations into the specific function of these genes might give insights into lineage specific developmental and morphogenetic differences.

## Methods

### Plant material

*Picea abies* L. Karst embryos and plants were grown as follows. Proliferating embryogenic cultures from cell line 28:05 were cultivated on solidified medium supplemented with the plant growth regulators (PGRs) auxin and cytokinin. To stimulate differentiation of early embryos the cultures were transferred to medium lacking PGRs and for further development of the embryos the cultures were transferred to medium supplemented with abscisic acid (ABA) [27]. Somatic embryo plants regenerated from cell line 28:05 were grown under accelerated growth conditions for seven growth periods in a phytotron [28]. Samples from the plants were collected at the end of the growth period during the second week (out of nine) under autumn growth conditions. Seedlings were derived from open pollinated seeds from a seed orchard germinated for two weeks in vermiculite at 22°C in micro-greenhouses under daylight conditions (approx. 16 h light).

Samples from nine different stages or tissues were collected for RNA extraction. Samples from embryogenic cultures were annotated as follows: proliferating Proembryogenic masses (PEM) in the presence of PGRs; Early embryos (EE) one week after withdrawal of PGRs; Late embryos (LE) two weeks after transfer to ABA containing medium; Mature cotyledonary embryos (ME) five weeks after transfer to ABA containing medium. The following tissues were collected from somatic embryo plants: shoot tips (ST), i.e. the topmost 2–3 mm of the elongating shoot; cambium (CM) dissected from branches where the bark was first removed; young needles, from elongating shoots (YN) and two year or older needles (ON). Root tips (RT) were collected from seedlings and the topmost 2–3 mm were sampled. The sampling was performed mid-day and all tissues were frozen in liquid nitrogen and stored at -80°C after collection.

### DNA and RNA isolation and cDNA synthesis

Genomic DNA was isolated from PEMs by DNeasy plant mini kit (Qiagen) or from needles from somatic embryo plants using CTAB (adapted from [29]). Total RNA was extracted from the samples described above using spectrum plant total RNA kit (Sigma) or RNeasy plant mini kit (Qiagen). DNase I (Sigma) was used to remove any genomic contamination. The RNA quality was checked by agarose gel electrophoresis and quantified by spectrophotometry. For each sample, 1 μg of total RNA was reverse transcribed with ReversAid H Minus First Strand cDNA Synthesis Kit (Fermentas) using 1:1 mixed random primer and oligo-dT primer (Fermentas) according to the manufacturer’s instructions.

### Cloning of *P. abies WOX* genes

*P*. *abies WOX* genes were identified by PCR using degenerate primers described in [30] as well as additional primers targeting the homeodomain in genomic DNA isolated from embryogenic cultures (Primer sequences can be found in Additional file 5). PCR-fragments of expected size (approx. 160 bp) were cloned using TOPO-kit from invitrogen and sequenced.

Each unique *WOX* homeodomain was extended by genome walking (5’ and 3’) using either Thermal Asymmetric Interlaced PCR (TAIL-PCR) [31] or Genome Walker Universal Kit (Clontech). Each gene was subsequently cloned using gene specific primers and sequenced. The gene structure was determined from the corresponding transcripts using gene specific primers or by 5’- and 3’ extension using 5’/3’ RACE technology (GeneRacer kit, Invitrogen). All cDNAs were cloned from PEM with the exceptions of *PaWOX3*, which was isolated from shoot tips (ST), and *PaWOX5*, which was isolated from root tips (RT). Gene predictions were performed with FGENESH (http://linux1.softberry.com/berry.phtml) trained with dicot plants (*Arabidopsis thaliana*). Primer sequences are listed in Additional file 5. Gene and cDNA accessions are listed in Additional file 6.

### Phylogenetic analysis

*WOX* gene sequences from *Ceratopteris*. *richardii* and *Cyathea australis* were the same as in [22] and *Nymphaea jamesoniana*, *Gnetum gnemon*, *Ginkgo biloba* and *Pinus sylvestris* were from [2]. Accession numbers for *Ostreococcus tauri*, *Ostreococcus lucimarinus*, *Physcomitrella patens*, *Selaginella moellendorffii*, *Oryza sativa* and *Vitis vinifera* can be found in Additional file 6.

Sequences were aligned using the translation alignment tool and the MAFFT plug-in in Geneious (Biomatters Ltd. Auckland, New Zealand) [32] and edited manually. For the large tree only the homeodomain was used, whereas additional sequences outside the homeodomain was used for the intermediate clade and modern clade sub-trees (alignments can be found in Additional file 4). Parts of the sequences that were difficult to align were excluded from the analyses to reduce noise. Furthermore, only genes for which the full-length sequence was known was used for the construction of the sub-trees.

Phylogenetic analyses were performed using both Bayesian inference (BI) and maximum likelihood (ML). For the BI analysis we used MrBayes (v. 3.1.2) [33]. The results were viewed using the MrBayes plug-in in Geneious. The nucleotide analyses were performed using the GTR model with a 4 category discrete gamma-distribution of rate variation among sites (GTR + G model). The data set was partitioned into three sets corresponding to codon position and rates were allowed to differ across partitions. The JTT model with gamma-distribution of rates among sites was chosen for the protein analysis with the aid of ProtTest 2.4 [34-36]. For the large tree the analysis was run for 10.000.000 generations with a burn-in of 1250. For the intermediate clade and modern clade sub-trees the analysis was run for 3.000.000 generations with a burn-in of 600 and 1250 respectively. The results from the intermediate clade runs were consistent despite the low burn-in (see Additional file 2). The ML analyses were performed using raxmlGUI 1.0 [37,38]. For nucleotide data the GTR gammacat model was used and for protein data we used the JTT + gamma model. Support was calculated using the rapid bootstrap method implemented in RAxML with 1000 bootstrap replicates. For the large tree the green alga sequence *OtWOX* was used as outgroup, whereas the fern sequence *CrWOXA* was used as outgroup for the intermediate clade and modern clade sub-trees.

### Expression analysis

The cDNA samples were amplified on an iQ5 Real-Time PCR Detection System (Bio-Rad). The reaction volume was 20 μl and Maxima SYBR green qPCR Master mix (Fermentas) was used. The three step cycling program was as follows: 95°C for 10 min, followed by 40 cycles at 95°C for 15 s, 58°C for 30s and 72°C for 30s. The melting curve analysis was conducted between 55°C-95°C. Four genes, *ELONGATION FACTOR*-*1* (*EFα*-*1*), *ACTIN*-*2* (*ACT2*), *UBIQUITIN* and *TUBULIN*-*9* were tested by geNorm (version 3.5, [39]) as candidate reference genes. PCR efficiencies of these reference candidates were decided by the linear regression method using the software LinRegPCR [40] and standard curve. No significant difference was found between these two methods. *EFα*-*1* and *ACT2* had the lowest stability M value. But as the efficiency of the PCR reaction of *ACT2* was lower than 1.85, only *EFα*-*1* was chosen as reference gene. PCR efficiencies of the eight target genes were decided by the linear regression method. Each target gene was run in nine different tissues with three technical replicates and two biological replicates. Wells showing strongly deviating PCR efficiencies of either target or reference gene were excluded from further analysis. Expression levels were calculated using the ΔΔCt method. Primer sequences are listed in Additional file 5. Accessions for reference genes are listed in Additional file 6.

## Competing interests

The authors declare that they have no competing interests.

## Authors’ contributions

HH did most of the cloning of genes and transcripts and participated in drafting of the manuscript. TZ did the qPCR analyses and part of the cloning. SvA participated in drafting of the manuscript and conceived the study. JS did the phylogenetic analyses, drafted the manuscript and conceived the study. All authors read and approved the final manuscript.

## Supplementary Material

Additional file 1 **Pairwise identity of the *P. abies* intermediate clade genes.** Pairwise identity is expressed as percent. Click here for file

Additional file 2 **Phylogenetic tree of the intermediate clade *WOX *genes including EST sequences from *Pinus taeda *(*Pt*).** Analyses were done using the BI method described in the methods sections. Exceptions were: amino acid model used was mixed, and burn-in was 1250. Support values are posterior probabilities DNA data above protein data. Click here for file

Additional file 3 **Amplification of *PaWOX2 *using the primers of Palovaara and Hakman [**[20]**] and the primers used in this study.** A. A schematic drawing of the *PaWOX2* locus showing the intron-exon pattern and the position of the primers and amplicons used by Palovaara and Hakman [20] (1) and in this study (2). Note that one of the primers in 2 binds at an intron-exon boundary. White box indicates the homeodomain. B. Results of qPCR analysis on the tissues used in this study using the primers of Palovaara and Hakman [20] and the primers used in this study. C. PCR using above mentioned primers on genomic DNA as well as cDNA. Click here for file

Additional file 4 Nucleotide and amino acid alignments used for the phylogenetic analyses. Click here for file

Additional file 5 **Primer sequences used for isolation, cloning and quantitative real-time PCR of *P. abies WOX* genes.** Click here for file

Additional file 6 **Accession numbers of *WOX *gene sequences isolated and used in this study.** Click here for file
