# Supplementary material for: Analysis of the WUSCHEL-RELATED HOMEOBOX gene family in the conifer picea abies reveals extensive conservation as well as dynamic patterns
Source: BMC Plant Biol. 2013 Jun 12;13:89. doi: 10.1186/1471-2229-13-89 (PMC3701499; doi:10.1186/1471-2229-13-89)
Supplement: Additional file 1 — Pairwise identity of the P. abies intermediate clade genes. Pairwise identity is expressed as percent. [file 1471-2229-13-89-S1.docx]

|  | *PaWOX8A* | *PaWOX8B* | *PaWOX8C* | *PaWOX8D* |
| --- | --- | --- | --- | --- |
| *PaWOX8/9* | 51.7 | 45.7 | 48.0 | 53.2 |
| *PaWOX8A* |  | 58.9 | 61.4 | 46.9 |
| *PaWOX8B* |  |  | 90.8 | 67.0 |
| *PaWOX8C* |  |  |  | 70.4 |

**Additional file 1.** Pairwise identity of the *P. abies* intermediate clade genes. Pairwise identity is expressed as percent.
