# Supplementary material for: Analysis of the WUSCHEL-RELATED HOMEOBOX gene family in the conifer picea abies reveals extensive conservation as well as dynamic patterns
Source: BMC Plant Biol. 2013 Jun 12;13:89. doi: 10.1186/1471-2229-13-89 (PMC3701499; doi:10.1186/1471-2229-13-89)
Supplement: Additional file 2 — Phylogenetic tree of the intermediate clade WOX genes including EST sequences from Pinus taeda (Pt). Analyses were done using the BI method described in the methods sections. Exceptions were: amino acid model used was mixed, and burn-in was 1250. Support values are posterior probabilities DNA data above protein data. [file 1471-2229-13-89-S2.pdf]

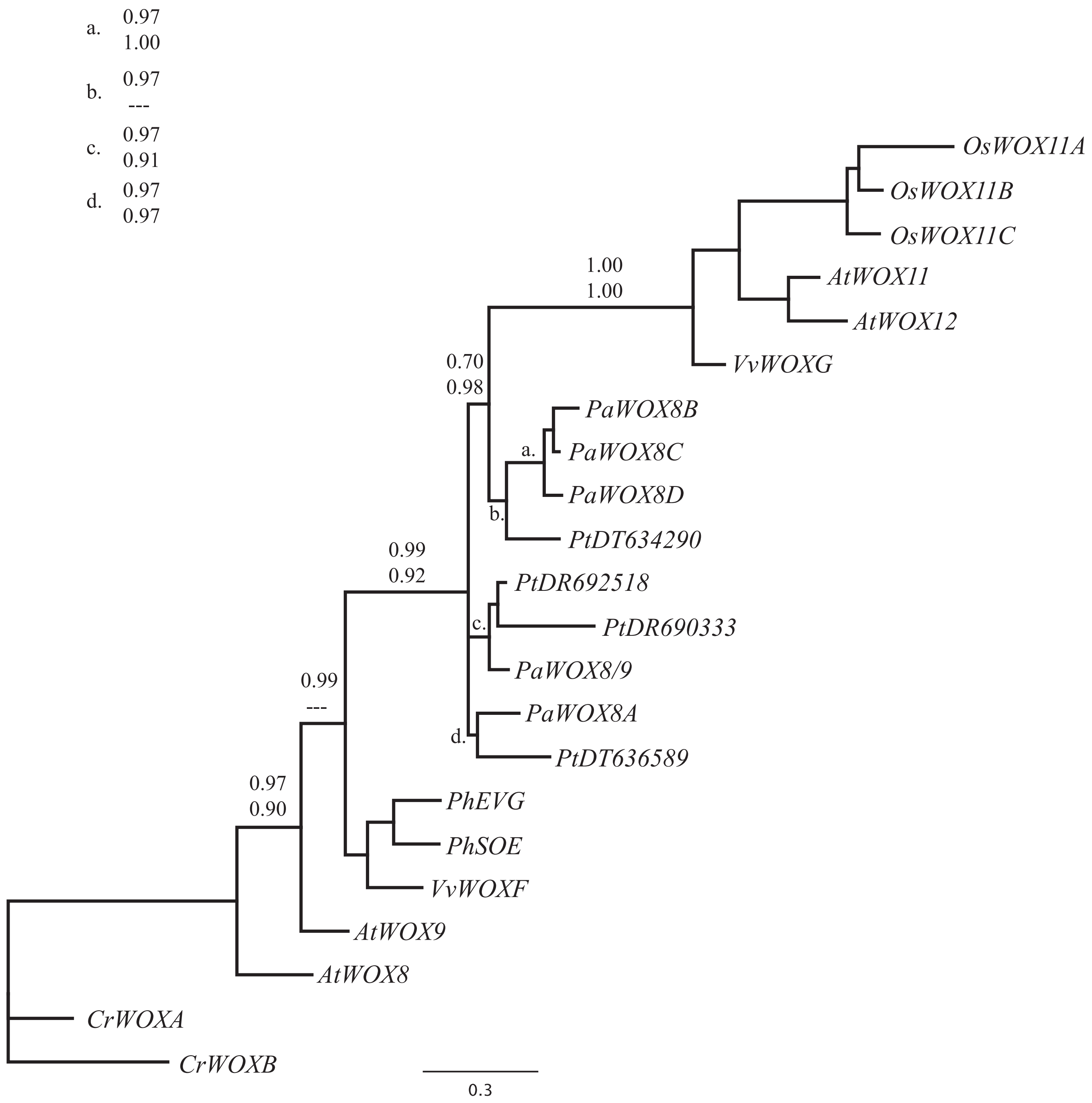

Additional file 2. Phylogenetic tree of the intermediate clade *WOX* genes including EST sequences from *Pinus taeda* (*Pt*). Analyses were done using the BI method described in the methods sections. Exceptions were: amino acid model used was mixed, and burn-in was 1250. Support values are posterior probabilities DNA data above protein data.

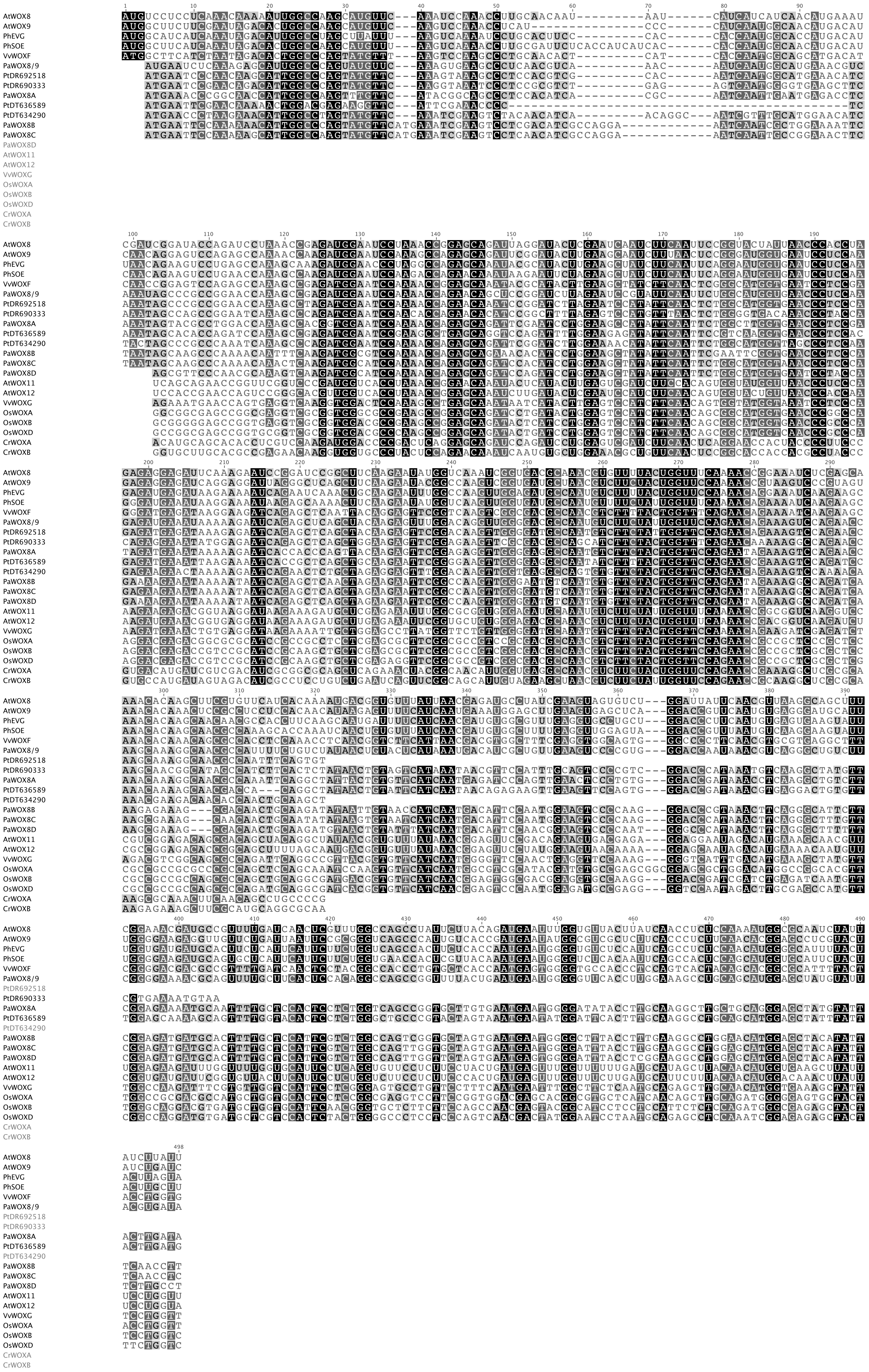
AtWOX8
AtWOX9
PhEVG
PhSOE
VvWOXF
PaWOX8/9
PtDR692518
PtDR690333
PaWOX8A
PtDT636589
PtDT634290
PaWOX8B
PaWOX8C
PaWOX8D
AtWOX11
AtWOX12
VvWOXG
OsWOXA
OsWOXB
OsWOXD
CrWOXA
CrWOXB

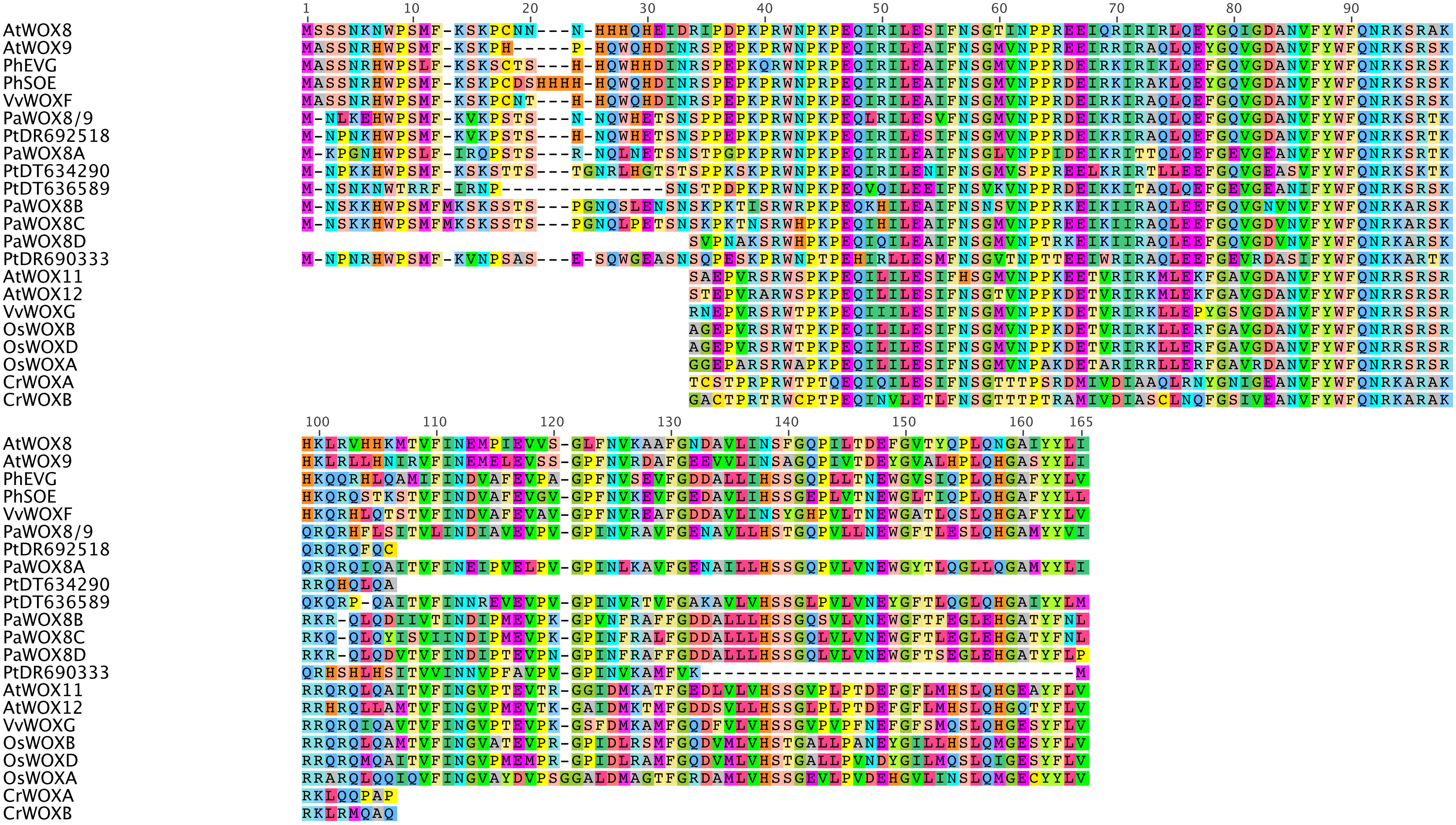
1 10 20 30 40 50 60 70 80 90
AtWOX8 MSSSNKNWPSMF-KSKPCNN---N-HHHQHEIDRIPDPKPRWNPKPEQIRILESIFNSGTINPPREEIQRIRIRLQEYGQIGDANVFYWFQNRKSRAK
AtWOX9 MASSNRHWPSMF-KSKPH------P-HQWQHDINRRSPEPKPRWNPKPEQIRILEAIFNSGMVNPPREEIRRIRAQLQEYGQVGDANVFYWFQNRKSRSK
PhEVG MASSNRHWPSLF-KSKSCTS---H-HQWHHDINRSPEPKQRWNPRPEQIRILEAIFNSGMVNPPRDEIRKIRIKLQEFGQVGDANVFYWFQNRKSRSK
PhSOE MASSNRHWPSMF-KSKPCDSHHHH-HQWQHDINRSPEPKPRWNPRPEQIRILEAIFNSGMVNPPRDEIRKIRAKLQEYGQVGDANVFYWFQNRKSRSK
VvWOXF MASSNRHWPSMF-KSKPCNT---H-HQWQHDINRSPEPKPRWNPKPEQIRILEAIFNSGMVNPPRDEIRKIRAQLQEFGQVGDANVFYWFQNRKSRSK
PaWOX8/9 M-NLKEHWPSMF-KVKPSTS---N-NQWHETSNSPPEPKPRWNPKPEQLRILESVFNSGMVNPPRDEIKRIRAQLQEFGQVGDANVFYWFQNRKSRTK
PtDR692518 M-NPNKHWPSMF-KVKPSTS---H-NQWHETSNSPPEPKPRWNPKPEQIRILESIFNSGMVNPPRDEIKRIRAQLQEFGQVGDANVFYWFQNRKSRTK
PaWOX8A M-KPGNHWPSLF-IRQPSTS---R-NQLNETSNSTPGPKPRWNPKPEQIRILEAIFNSGLVNPPIDEIKRITTQLQEFGEVGEANVFYWFQNRKSRTK
PtDT634290 M-NPKKHWPSMF-KSKSTTS---TGNRLHGTSTSPPKSKPRWNPKPEQIRILENIFNSGMVSPPREELKRIRTLLEEFGQVGEASVFYWFQNRKSKTK
PtDT636589 M-NSNKNWTRRF-IRNP----------------SNSTPDPKPRWNPKPEQVQILEEIFNSVKVNPPRDEIKKITAQLQEFGEVGEANIFYWFQNRKSRSK
PaWOX8B M-NSKKHWPSMFMKSKSSTS---PGNQSLENSNSKPKTISRWRPKPEQKHILEAIFNSNSVNPPRKEIKIIRAQLEEFGQVGNVNVFYWFQNRKARSK
PaWOX8C M-NSKKHWPSMFMKSKSSTS---PGNQLPETSNSKPKTNSRWHPKPEQIHILEAIFNSGMVNPPREEIKIIRAQLEEFGQVGDVNVFYWFQNRKARSK
PaWOX8D SVPNAKSRWHPKPEQIQILEAIFNSGMVNPTRKEIKIIRAQLEEFGQVGDVNVFYWFQNRKARSK
PtDR690333 M-NPNRHWPSMF-KVNPSAS---E-SQWGEASNSQPESKPRWNPTPEHIRLLESMFNSGVTNPTTEEIWRIRAQLEEFGEVRDASIFYWFQNKKARTK
AtWOX11 SAEPVRSRWSPKPEQILILESIFHSGMVNPPKEETVRIRKMLEKFGAVGDANVFYWFQNRRSRSR
AtWOX12 STEPVRARWSPKPEQILILESIFNSGTVNPPKDETVRIRKMLEKFGAVGDANVFYWFQNRRSRSR
VvWOXG RNEPVRSRWTPKPEQIIILESIFNSGMVNPPKDETVRIRKLLEPYGSVGDANVFYWFQNRRSRSR
OsWOXB AGEPVRSRWTPKPEQILILESIFNSGMVNPPKDETVRIRKLLERFGAVGDANVFYWFQNRRSRSR
OsWOXD AGEPVRSRWTPKPEQILILESIFNSGMVNPPKDETVRIRKLLERFGAVGDANVFYWFQNRRSRSR
OsWOXA GGEPARSRWAPKPEQILILESIFNSGMVNPAKDETARIRRLLERFGAVRDANVFYWFQNRRSRSR
CrWOXA TCSTPRPRWTPTQEQIQILESIFNSGTTTPSRDMIVDIAAQLRNYGNIGEANVFYWFQNRKARAK
CrWOXB GACTPRTRWCPTPEQINVLETLFNSGTTTPTRAMIVDIASCLNQFGSIVEANVFYWFQNRKARAK
100 110 120 130 140 150 160 165
AtWOX8 HKLRVHHKMTVFINEMPIEVVS-GLFNVKAAFGNDAVLINSFGQPILTDEFGVTYQPLQNGAIYYLI
AtWOX9 HKLRLLHNIRVFINEMELEVSS-GPFNVRDAFGEEVVLINSAGQPIVTDEYGVALHPLQHGASYYLI
PhEVG HKQQRHLQAMIFINDVAFEVPA-GPFNVSEVFGDDALLIHSSGQPLLTNEWGVSIQPLQHGAFYYLV
PhSOE HKQRQSTKSTVFINDVAFEVGV-GPFNVKEVFGEDAVLIHSSGEPLVTNEWGLTIQPLQHGAFYYLL
VvWOXF HKQRHLQTSTVFINDVAFEVAV-GPFNVREAFGDDAVLINSYGHPVLTNEWGATLQSLQHGAFYYLV
PaWOX8/9 QRQRHFLSITVLINDIAVEVPV-GPINVRAVFGENAVLLHSTGQPVLLNEWGFTLESLQHGAMYYVI
PtDR692518 QRQRQFQC
PaWOX8A QRQRQIQAITVFINEIPVELPV-GPINLKAVFGENAILLHSSGQPVLVNEWGYTLQGLLQGAMYYLI
PtDT634290 RRQHQLQA
PtDT636589 QKQRP-QAITVFINNREVEVPV-GPINVRTVFGAKAVLVHSSGLPVLVNEYGFTLQGLQHGAIYYLM
PaWOX8B RKR-QLQDIIVTINDIPMEVPK-GPVNFRAFFGDDALLLHSSGQSVLVNEWGFTFEGLEHGATYFNL
PaWOX8C RKQ-QLQYISVIINDIPMEVPK-GPINFRALFGDDALLLHSSGQLVLVNEWGFTLEGLEHGATYFNL
PaWOX8D RKR-QLQDVTVFINDIPTEVPN-GPINFRAFFGDDALLLHSSGQLVLVNEWGFTSEGLEHGATYFLP
PtDR690333 QRHSHLHSITVVINNVPFAVPV-GPINVKAMFVK-------------------------------M
AtWOX11 RRQRQLQAITVFINGVPTEVTR-GGIDMKATFGEDLVLVHSSGVPLPTDEFGFLMHSLQHGEAYFLV
AtWOX12 RRHRQLLAMTVFINGVPMEVTK-GAIDMKTMFGDDSVLLHSSGLPLPTDEFGFLMHSLQHGQTYFLV
VvWOXG RRQRQIQAVTVFINGVPTEVPK-GSFDMKAMFGQDFVLVHSSGVPVPFNEFGFSMQSLQHGESYFLV
OsWOXB RRQRQLQAMTVFINGVATEVPR-GPIDLRSMFGQDVMLVHSTGALLPANEYGILLHSLQMGESYFLV
OsWOXD RRQRQMQAITVFINGVPMEMPR-GPIDLRAMFGQDVMLVHSTGALLPVNDYGILMQSLQIGESYFLV
OsWOXA RRARQLQQIQVFINGVAYDVPSGGALDMAGTFGRDAMLVHSSGEVLPVDEHGVLINSLQMGECYYLV
CrWOXA RKLQQPAP
CrWOXB RKLRMQAQ
