# Supplementary material for: Analysis of the WUSCHEL-RELATED HOMEOBOX gene family in the conifer picea abies reveals extensive conservation as well as dynamic patterns
Source: BMC Plant Biol. 2013 Jun 12;13:89. doi: 10.1186/1471-2229-13-89 (PMC3701499; doi:10.1186/1471-2229-13-89)
Supplement: Additional file 3 — Amplification of PaWOX2 using the primers of Palovaara and Hakman [[20]] and the primers used in this study. A. A schematic drawing of the PaWOX2 locus showing the intron-exon pattern and the position of the primers and amplicons used by Palovaara and Hakman [20] (1) and in this study (2). Note that one of the primers in 2 binds at an intron-exon boundary. White box indicates the homeodomain. B. Results of qPCR analysis on the tissues used in this study using the primers of Palovaara and Hakman [20] and the primers used in this study. C. PCR using above mentioned primers on genomic DNA as well as cDNA. [file 1471-2229-13-89-S3.pdf]

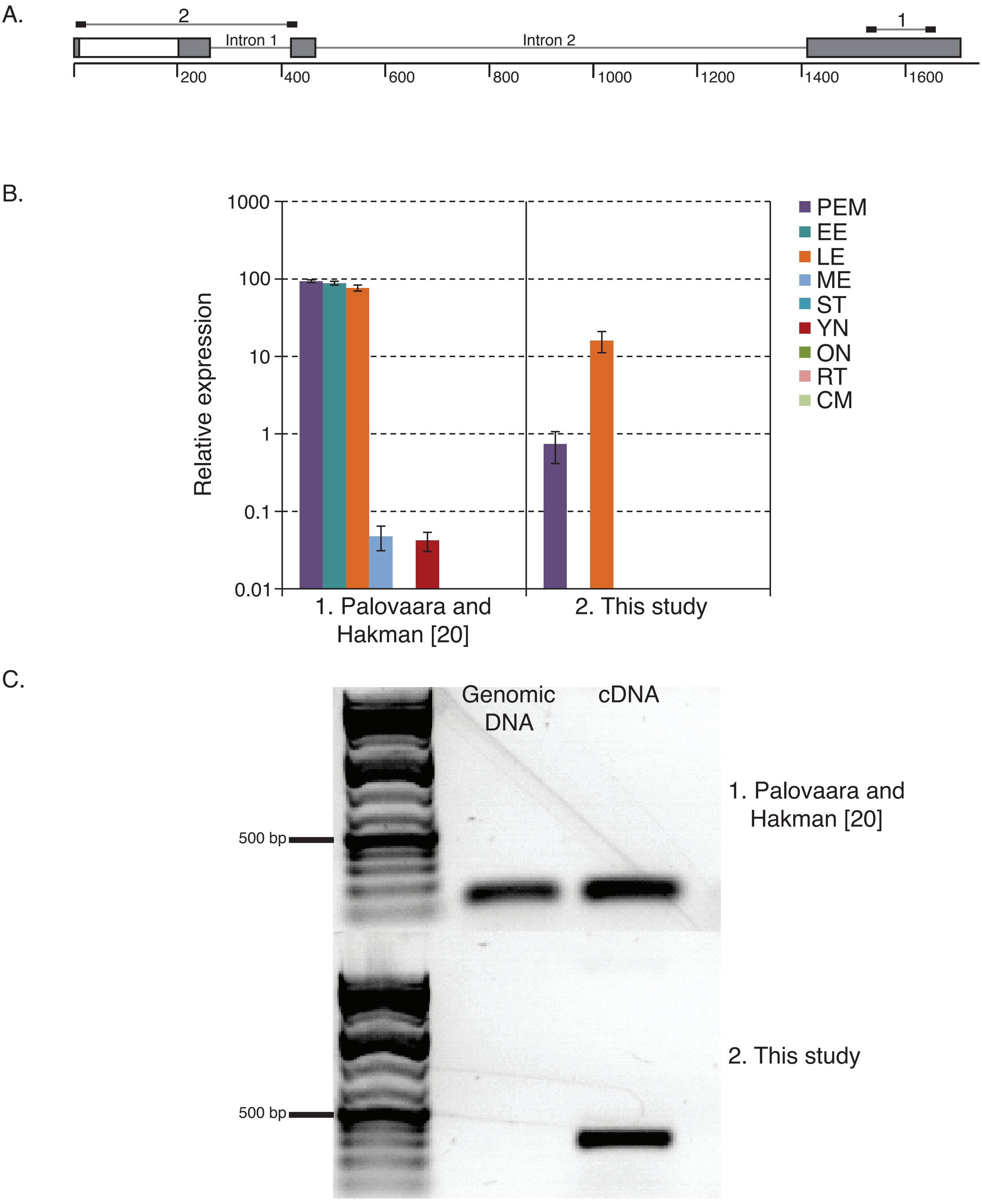

Amplification of *PaWOX2* using the primers of Palovaara and Hakman [20] and the primers used in this study. A. A schematic drawing of the *PaWOX2* locus showing the intron-exon pattern and the position of the primers and amplicons used by Palovaara and Hakman [20] (1) and in this study (2). Note that one of the primers in 2 binds at an intron-exon boundary. White box indicates the homeodomain. B. Results of qPCR analysis on the tissues used in this study using the primers of Palovaara and Hakman [20] and the primers used in this study. C. PCR using above mentioned primers on genomic DNA as well as cDNA.
