# Supplementary material for: Analysis of the WUSCHEL-RELATED HOMEOBOX gene family in the conifer picea abies reveals extensive conservation as well as dynamic patterns
Source: BMC Plant Biol. 2013 Jun 12;13:89. doi: 10.1186/1471-2229-13-89 (PMC3701499; doi:10.1186/1471-2229-13-89)
Supplement: Additional file 4 — Nucleotide and amino acid alignments used for the phylogenetic analyses. [file 1471-2229-13-89-S4.pdf]

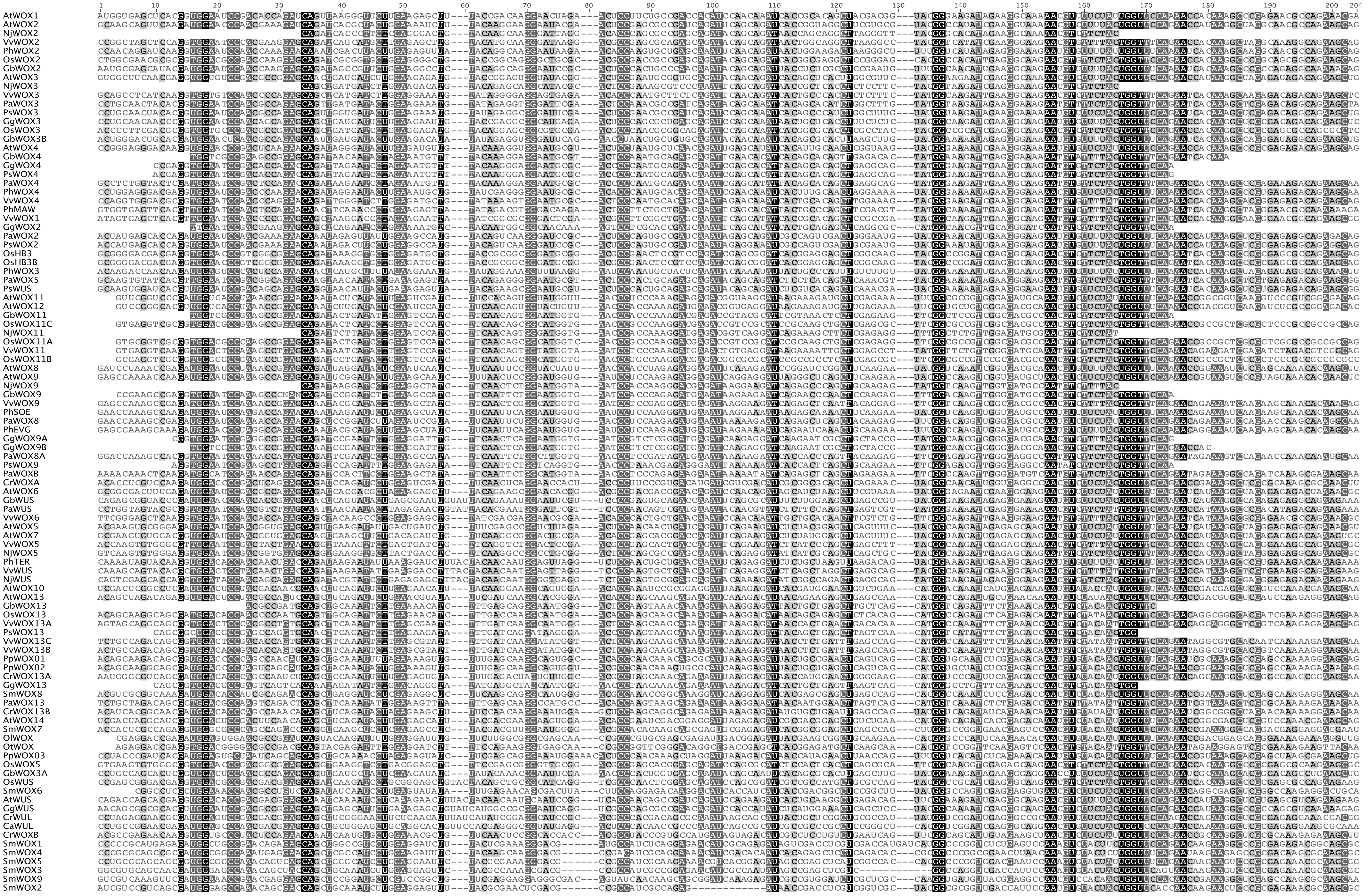

Nucleotide aligment used for the construction of the full *WOX* gene tree.

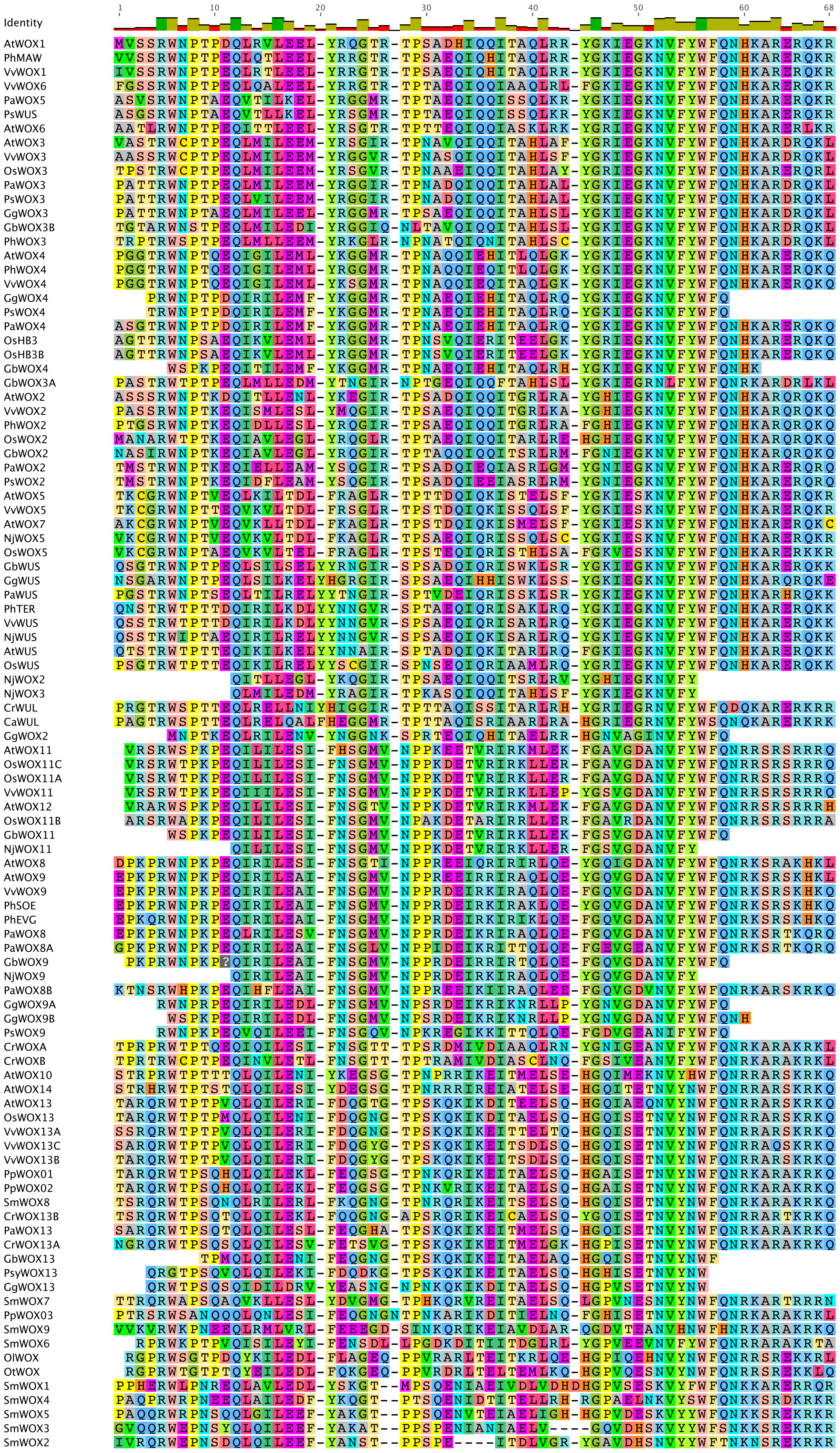

Amino acid alignment used for the construction of the full WOX gene tree.

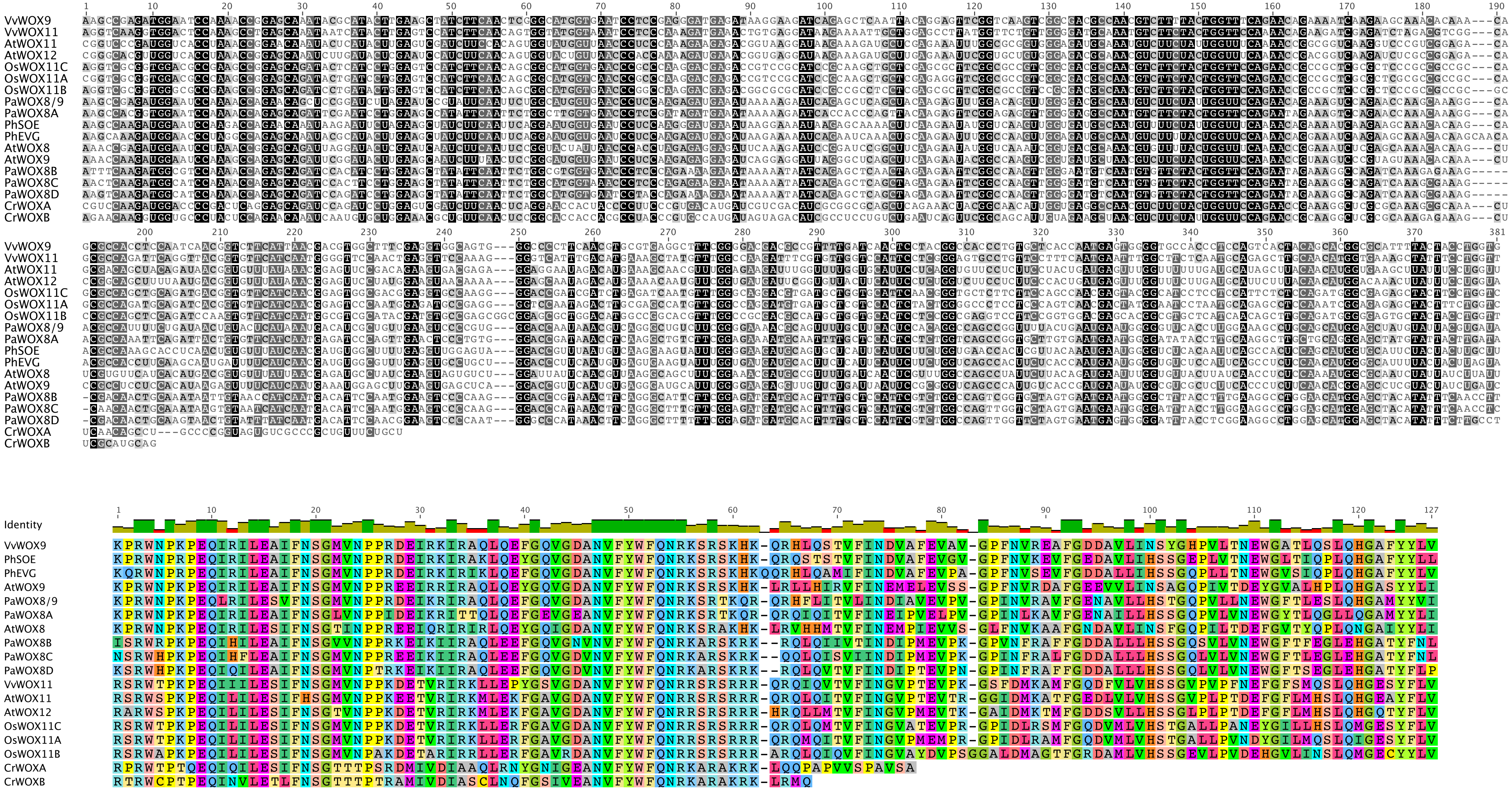

Nucleotide and amino acid alignments used for the construction of the intermediate clade WOX gene tree.

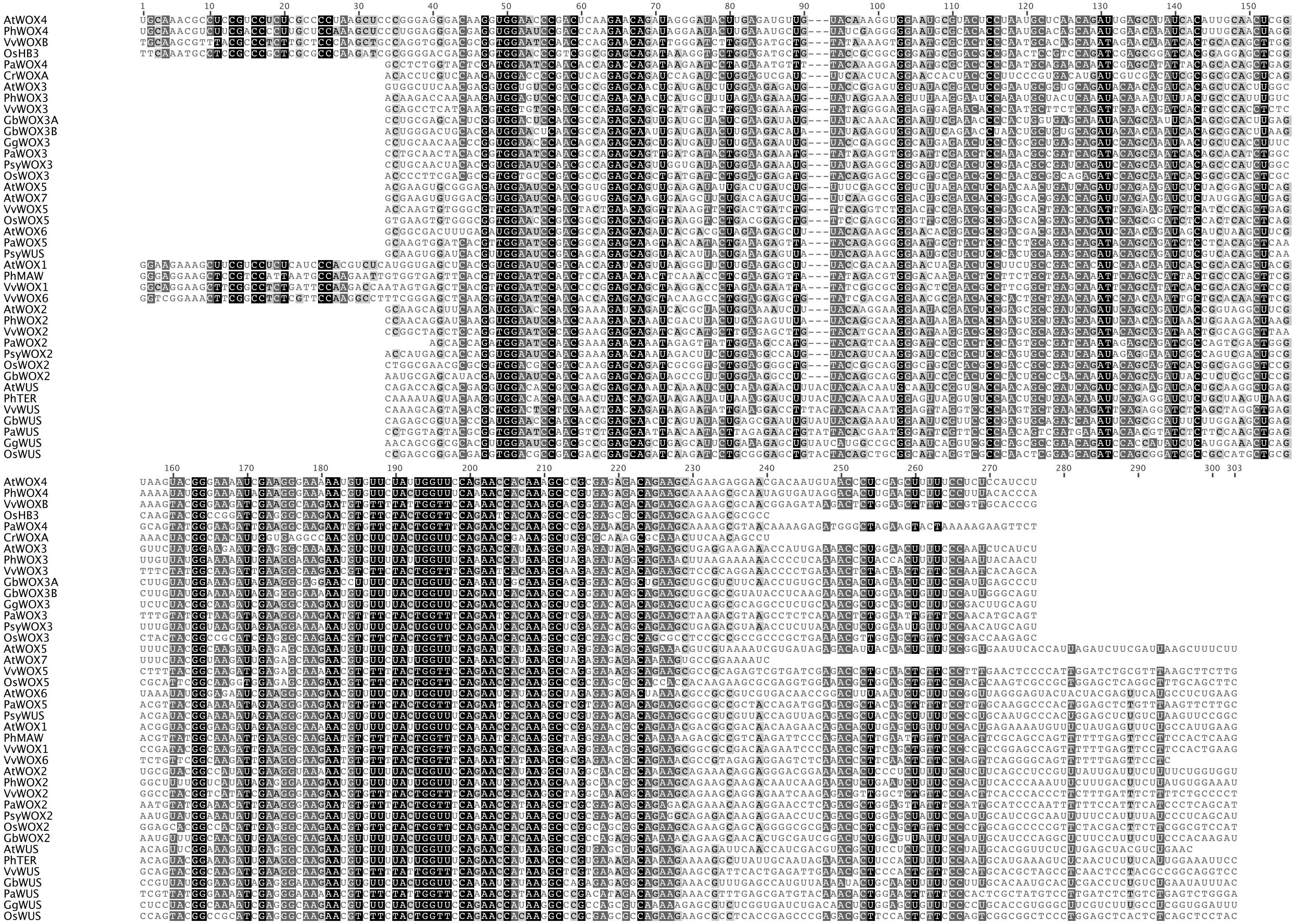

Nucleotide Alignment used for the construction of the modern clade *WOX* gene tree.

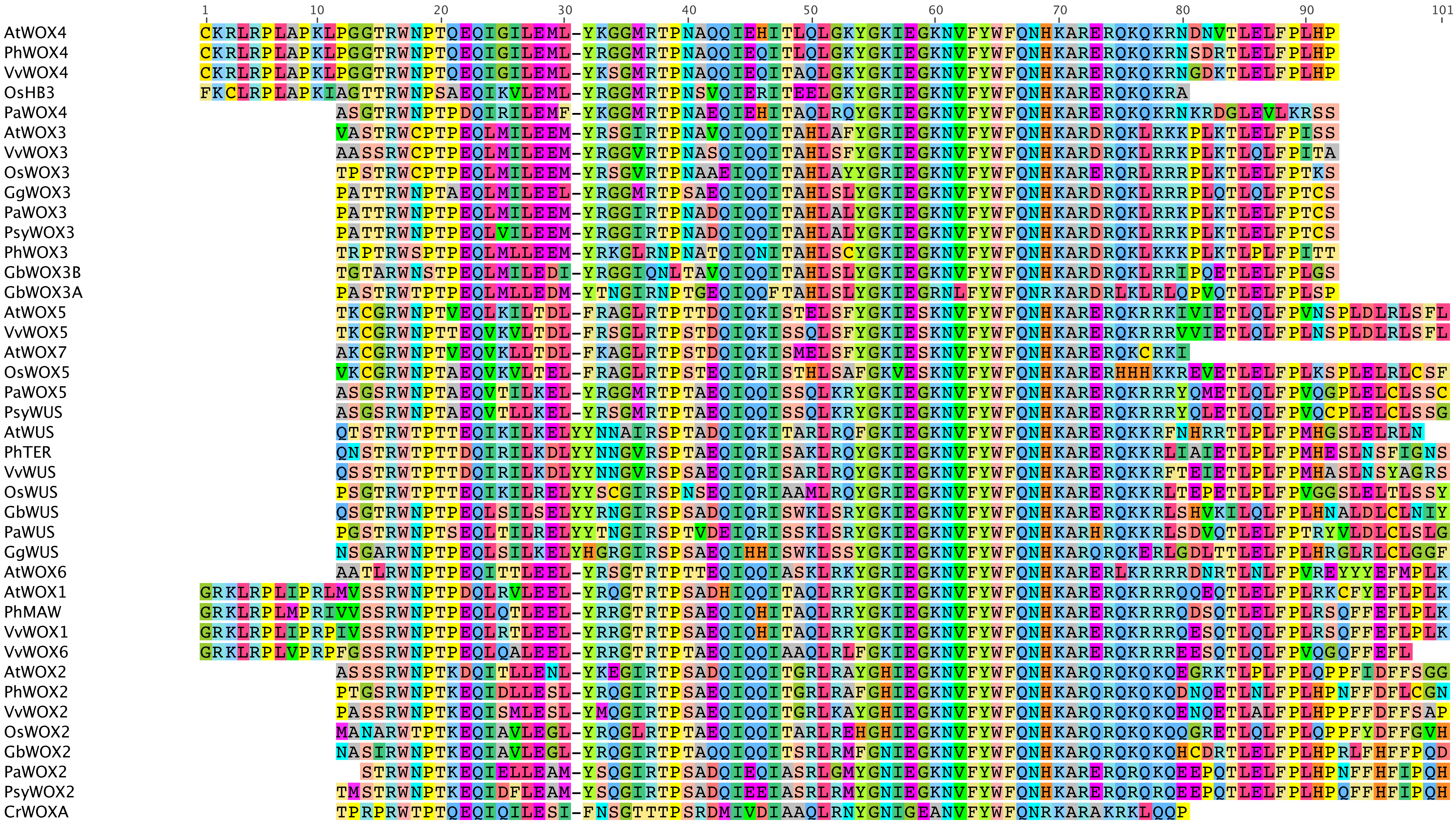

Amino acid alignment used for the construction of the modern clade *WOX* gene tree.
