# Supplementary material for: Analysis of the WUSCHEL-RELATED HOMEOBOX gene family in the conifer picea abies reveals extensive conservation as well as dynamic patterns
Source: BMC Plant Biol. 2013 Jun 12;13:89. doi: 10.1186/1471-2229-13-89 (PMC3701499; doi:10.1186/1471-2229-13-89)
Supplement: Additional file 6 — Accession numbers of WOX gene sequences isolated and used in this study. [file 1471-2229-13-89-S6.docx]

**Accession numbers of *WOX* gene sequences isolated and used in this study.**

| **Name** | **Accession** | **Name** | **Accession** | **Name** | **Accession** |
| --- | --- | --- | --- | --- | --- |
| *OtWOX* | XM_003082558 | *OsHB3* | AJ556181 | *VvWOX6* | LOC100250994 |
| *OlWOX* | XM_001420899 | *OsHB3B* | Os04g0649400 | *VvWOX9* | LOC100258905 |
| *PpWOX01* | XM_001777582 | *OsWOX2* | Os01g0840300 | *VvWOX11* | LOC100246660 |
| *PpWOX02* | XM_001757640 | *OsWOX3* | AB218893 | *VvWOX13A* | LOC100251229 |
| *PpWOX03* | XM_001777125 | *OsWOX5* | Os01g0854500 | *VvWOX13B* | LOC100267830 |
| *SmWOX1* | XM_002965891 | *OsWOX11A* | Os03g0325600 | *VvWOX13C* | LOC100248809 |
| *SmWOX2* | XM_002962345 | *OsWOX11B* | Os08g0242400 | *PaWOX3* | JX411947 |
| *SmWOX3* | XM_002962343 | *OsWOX11C* | Os07g0684900 | *PaWOX4* | JX411948 |
| *SmWOX4* | XM_002977711 | *OsWOX13* | Os01g0818400 | *PaWOX5* | JX411949 |
| *SmWOX5* | XM_002977608 | *VvWUS* | LOC100267850 | *PaWOX8A* | JX411950 |
| *SmWOX6* | XM_002981839 | *VvWOX1* | LOC100250993 | *PaWOX8B* | JX411951 |
| *SmWOX7* | XM_002964019 | *VvWOX2* | LOC100263784 | *PaWOX8C* | JX411952 |
| *SmWOX8* | XM_002962367 | *VvWOX3* | LOC100260450 | *PaWOX8D* | JX411953 |
| *SmWOX9* | XM_002965896 | *VvWOX4* | LOC100250666 | *PaWUS* | JX512364 |
| *OsWUS* | AB218894 | *VvWOX5* | LOC100258187 |  |  |
